# Health utility of children with acute lymphoblastic leukemia in China

**DOI:** 10.3389/fpubh.2022.1069336

**Published:** 2023-01-04

**Authors:** Wei Wang, Yaqi Dong, Mingjing Ji, Xiaoyan Zhang, Jiaoyang Cai

**Affiliations:** ^1^Key Lab of Health Technology Assessment, National Health Commission of the People's Republic of China (Fudan University), School of Public Health, Fudan University, Shanghai, China; ^2^Key Laboratory of Pediatric Hematology and Oncology of China Ministry of Health, Department of Hematology and Oncology, Shanghai Children's Medical Center, Shanghai Jiao Tong University School of Medicine, Shanghai, China

**Keywords:** acute lymphoblastic leukemia, children, quality of life, health utility, outcome research, treatment phrase

## Abstract

**Background:**

Acute lymphoblastic leukemia is the most common cancer in children. As the 5-year survival rate has been improved to over 80%, more emphasis is now placed on reducing therapy toxicities and enhancing health-related quality of life (HRQoL) of patients during treatment. Our objective was to measure health utility of pediatric acute lymphoblastic leukemia (pALL) patients in China, examine utility weights of different treatment phases and influencing factors of health utility, as well as identify which aspects of HRQoL were most impaired.

**Methods:**

A cross-sectional study was conducted in Shanghai Children's Medical Center (SCMC) Affiliated to Shanghai Jiao Tong University School of Medicine in China from April to November 2021. Primary caregivers of 247 patients completed the assessment by CHU9D-CHN and health utility scores were computed for all the patients and stratified by treatment phases. Various multivariable models were constructed and the best was chosen to identify independent factors associated with utility scores. Factors affecting the most impaired dimensions were also examined.

**Results:**

The overall mean (SD) health utility score was 0.79 (±0.17) and significantly increased from induction (0.73 [±0.19], *P* < 0.001) to consolidation (0.74 [±0.18]), and to maintenance (0.82 [±0.16]). After adjusting for potentially influencing factors, utility scores in induction (*Beta* = −0.086, *P* = 0.005) and consolidation (*Beta* = −0.074, *P* = 0.043) were constantly lower than those during maintenance. In item-level analysis, lower age and induction phase were found to be significantly associated with high severity reported on the “school work/homework” dimension. Additionally, only the induction phase (vs. maintenance, OR = 2.24, *P* = 0.016) was independently associated with the high severity level reported on the “able to join in activities” dimension.

**Conclusions:**

This is the first study that measured health utility of children with pALL in China. Mean health utility scores increased from induction to maintenance. These provided important utility estimates that help inform future health economic models. The phrasing of “School work/homework” in CHU9D-CHN could be further improved. More efforts are needed to design and implement specific interventions targeting at the dimension “able to join in activities” for enhancing HRQoL of children with pALL in China.

## Introduction

Acute lymphoblastic leukemia is the most common cancer in children, accounting for about 26% of cancers diagnosed between the ages of 0 and 14 years (1). Because of advances in early diagnosis and treatment, the 5-year survival rate of pediatric acute lymphoblastic leukemia (pALL) can be over 80% (2). While new types of treatment are being tested in clinical trials such as targeted therapy and immunotherapy, chemotherapy remains the standard treatment for newly diagnosed pALL patients (3, 4). The chemotherapy usually has three phases–induction, consolidation and maintenance, reflecting a reliance on sequential multidrug regimens of varying intensity to avoid development of resistance: Remission induction is the first phase of chemotherapy, lasting 4 to 6 weeks. Patients are usually admitted to a hospital for initial treatment and workup; Consolidation aims to eradicate the submicroscopic residual disease that remains after a complete remission is obtained; Maintenance is the final and longest phase with a much less intensive regimen than the prior chemotherapy (3, 5). Each phase may vary in exact length and intensity among different treatment protocols, but they all shared the same principle of chemotherapy for pALL: to provide phased and long-term standardized treatment with intensive chemotherapy in the early phase, followed by less intensive treatment (4). Today, more emphasis has been placed on reducing the toxicities of chemotherapy, focusing on the health status and quality of life of patients during the treatment.

Health utility is a quantitative measurement of health-related quality of life (HRQoL) and describes an individual's preference for a health state on a scale from 0 to 1, where 0 represents death and 1 indicates full health status with negative values assigned to states worse than death (6). It has been an essential element in the value frameworks of the American Society of Clinical Oncology (7), and also a crucial part of cost utility analysis which is now strongly recommended for clinical comprehensive evaluation of drugs and pharmacoeconomic evaluation according to two recent Chinese guidelines (8, 9). In health economic evaluation (HEE) models for pALL (10, 11), treatment phases were commonly used to define different health states. For example, Health Quality Ontario (12) set health states according to 4 treatment phases (i.e., induction, consolidation, intensification, and maintenance), while Lin et al. (13) added another health state “continuation” between intensification and maintenance as per a different treatment protocol. For each treatment phase, an accurate estimate of health utility is required to calculate quality-adjusted life years (QALYs) that facilitate comparison of the cost-effectiveness of health interventions from diverse areas.

Despite the significant value of health utility for a HEE model based on pALL treatment phases, very few studies measured health utilities of children with pALL at different treatment phases and all reported significant differences among treatment phases. In fact, only 4 relevant studies were identified from an updated search built on two recent reviews [one in 2017 by Fardell et al. (11) and the other in 2021 by Chen et al. (10)]. All were longitudinal studies using HUI2 and/or HUI3. One of them reported an improvement in general HRQoL, measured by the HUI3, in the first 12 months of treatment (14). The other three studies were from the same research team by Furlong et al. and showed a common trend that utility scores generally increased from induction to the post-treatment phase (15–17), despite differences in their treatment protocols and study periods. Since they are known to be influenced by varying socioeconomic and cultural backgrounds, it is not ideal to adopt health utility scores from overseas populations (18). Besides, considering potential differences in clinical practices of treating pALL, it is not clear whether the trend would remain for children with pALL in China. To the best of our knowledge, no study has yet reported health utilities of children with pALL at different treatment phases, or examined the association between pALL health utility values and treatment phases in China.

Methodologically, out of 9 multi-attribute utility instruments available for use in children and adolescents worldwide, CHU9D is the only utility instrument that was developed from its inception with young people (19). It contains 9 questions with child-friendly phrasing and each asks how respondents feel today in a distinct aspect (20). The most important reason for choosing it for this study is that only CHU9D contains a China-specific value set which is used as a scoring algorithm for converting CHU9D responses to utility scores (21). The Chinese version of CHU9D (i.e., CHU9D-CHN) was developed in line with ISPOR Task Force for translation and cultural adaptation (22) and found to be a satisfactory, reliable, and valid instrument to measure and value HRQoL for healthy children and adolescents in China (23, 24).

In view of the above, our study aims to measure health utility values of pALL patients in China with CHU9D-CHN, to evaluate utility weights of different treatment phases, to explore influencing factors of health utility, and to identify which aspect(s) of HRQoL were most impaired. The finding may add valuable information to the scarce evidence base and inform QALYs estimation in model-based health economic evaluations for allocation and prioritization of scarce healthcare resources for China. It may also help design targeted interventions to improve HRQoL of children with pALL.

## Materials and methods

### Subjects and study design

In this cross-sectional study, convenience sampling was used. Consecutive patient-caregiver dyads seen at the Shanghai Children's Medical Center (SCMC) Affiliated to Shanghai Jiao Tong University School of Medicine, Shanghai, China between April and November 2021 were recruited. To maximize our sample size, we also included dyads from an early pilot run in November 2019. The inclusion criteria of the patients were: Chinese residents who had a confirmed diagnosis of ALL were undergoing formal clinical treatment at the center, and were aged 4 to 18 years on the day of survey. All the eligible patients were undergoing treatments according to the Chinese Children's Cancer Group study ALL-2015 (CCCGALL-2015) protocol (25, 26). CCCGALL-2015 was a minimal residual disease (MRD)-directed, risk-stratified treatment protocol for newly diagnosed pALL, which has been widely adopted by 20 major hospitals/medical centers in 10 provinces, three central government direct-controlled municipalities and Hong Kong since 1 January 2015 (25, 26). The catchment areas of these centers contain approximately 65% of the population of China. CCCGALL-2015 comprises three treatment phases—induction (week 1–7), consolidation (week 8–15) and maintenance (week 16–125). In the early part (from week 16 to week 35) of the maintenance phase, patients undergo different continuation therapy according to their risk status, while from week 36–125 patients receive pulse therapy with dexamethasone and vincristine during maintenance therapy [details about the regimens were available in a published clinical paper (26)].

Accompanying primary informal caregivers of the eligible patients were surveyed to assess health utility values of the patients. Primary informal caregivers were defined as the family member who are most involved in providing care or ensuring provision of care to the patient. Participants who could not understand or read Chinese were excluded. Written informed consent was obtained from all participants. This study was reviewed and approved by the hospital ethical Review Board. Written informed consent was obtained from the parents, guardians, or patients, as appropriate.

### Data collection

#### Socio-demographic and clinical data

Data were mainly retrieved from a prospective clinical database, which comprised data of initial and subsequent follow-up outpatient visits and hospitalization episodes of all pALL patients managed at the SCMC since inception. Informed consent was obtained from patients' legal guardians for their data to be included in the database as per institutional ethics board requirements. The socio-demographic data retrieved included patients' age, gender, urban residents (yes vs. no), living region (from vs. outside Shanghai), and annual disposable household income per capita level. Clinical data retrieved included co-morbid diseases, disease severity (intermediate/high risk vs. low), presence of serious adverse events in the preceding 4 weeks (yes vs. no), use of steroids today or in the preceding week (yes vs. no), and disease duration since diagnosis. Disease severity was represented by patient risk status which was determined by the leukemia molecular subtype and minimal residual disease [criteria for the classification of risk groups are described in the CCCGALL-2015 protocol (25)].

The income level was obtained from a pre-defined survey questionnaire to collect socio-demographic information of primary caregivers due to the lack of complete information in the database. The questionnaire consists of 10 items asking primary caregivers' age, relationship to child, number of children, etc. It was pretested with CHU9D-CHN in the pilot run of 10 patient-parent dyads to ensure all the questions were easy to understand. Information other than annual disposable household income per capita level is reserved for a separate analysis and thus was not reported in this study. The questionnaire and CHU9D-CHN were self-administered *via* paper and pencil by each consenting respondent in the outpatient clinic or wards of the SCMC hematological oncology department, with onsite assistance provided from a trained research nurse whenever needed. Two researchers conducted independently data entry of paper-based questionnaires and data extraction from the clinical database. Any discrepancy was resolved through either discussion with the respondent or verification against information documented in medical records.

#### Utility data

CHU9D is suitable for use in children and adolescents aged 7–17 years (27). The CHU9D-CHN consists of 9 items covering the following 9 dimensions–worry, sad, pain, tired, annoyed, school work/homework, sleep, daily routine, able to join in activities. Each item contains five response levels presenting increasing degrees of severity within each dimension. The recall period is “today”. The Chinese value set of CHU9D was derived from 923 students aged 9–17 years using the Best-Worst Scaling (BWS) and time trade-off (TTO) methods (18). It is known that self-reported utility scores are always preferred in theory, however, in our study, about 56% of children with pALL were < 8 years old and didn't have adequate reading and comprehension abilities to complete the survey (28). Therefore, this study presented the CHU9D-CHN responses that were assessed by accompanying primary informal caregivers, i.e., proxy-rated utility scores.

### Statistical analysis

Descriptive statistics of patient characteristics and utility scores were presented and stratified by treatment phases (i.e., induction, consolidation, and maintenance). Continuous variables were presented as mean and standard deviation (SD), while categorical variables were shown as frequency and percentage. Differences among treatment phases were assessed using Fisher exact test for categorical variables and ANOVA or Kruskal–Wallis test for normally and non-normally distributed continuous variables, respectively. Next, bivariate analysis was conducted to identify potentially influencing factors of the utility scores, and the variables were selected for testing according to expected clinical relevance and findings from the previous studies.

Multivariable regression models were built to identify independent factors (such as age, gender, treatment phase etc.) that were associated with utility scores. For scores with highly skewed distribution, we used robust standard error estimates. To explore the independent effects of treatment phase after adjusting for different sets of confounding factors, stepwise multivariate linear regression models were first constructed whereby the independent variables were entered in the following steps: Step 1: treatment phases were entered in Model 1; Step 2: adjusted factors including age, gender, disease severity, and income level were added to Model 2, Model 3, Model 4, and Model 5, respectively. To check whether the ordinary least square (OLS) model was appropriate, the linearity of residuals, homogeneity of variance, collinearity, high leverage points, and normality of residuals were examined by plots using *performance* (V0.9.0) package in *R* (V4.1.0).

For model comparison, other types of models were also considered: general linear models (GLM) with various combinations of link functions and family distributions, Tobit model, and two-part model. The latter two models were usually used to analyze utility data with ceiling effects. Model diagnostics check was run to ensure model assumptions were satisfied for each type of model. The OLS model was chosen as our final model as it has the lowest Akaike Information Criteria (AIC).

The distribution of responses to each CHU9D item was presented as frequency and percentage. We combined the 5-level response to form a binary variable of high severity level (the two most severe degrees) vs. low severity level (the other 3 degrees). For dimensions with more than 30% of respondents reporting high severity level, Fisher exact test and Student's *t*-test were used to investigate the association of responses to each dimension with categorical participant characteristics and continuous ones respectively. Multivariable logistic regression models were also built to explore independent factors (such as age, gender, treatment phase etc.) that were associated with responses to the CHU9D dimensions which were most impaired.

All tests of significance were two-sided, and *P* < 0.05 was considered to indicate statistical significance. Analyses were performed by *R* version 4.1.0.

## Results

### Characteristics of participants

Primary caregivers of 247 eligible patients completed the survey with a response rate of 95.4%. At the day of survey, 21% of the patients were at the induction phase, 11% at the consolidation, and 68% at the maintenance. Table 1 presents patient demographic, socioeconomic, and clinical characteristics stratified by treatment phases. The mean (SD) age of patients was 8.11 (±3.47), 56.3% were male and 50.2% are urban residents. Most patients (198 [80.2%]) were not from Shanghai and reported annual disposable household income level above 50,000 CNY per capita (172 [69.6%]).

**Table 1:** Demographic and socioeconomic characteristics of patients by treatment phases.

**Characteristics**	**Total** **(*N* =247)**	**Treatment phases**			***P*** **-value^a^**
		**Induction** **(*****N*** = **53)**	**Consolidation** **(*****N*** = **26)**	**Maintenance** **(*****N*** = **168)**	
**Age (years)**					0.124
Mean (SD)	8.11 (±3.47)	7.33 (±3.00)	7.80 (±3.74)	8.41 (±3.54)	
**Gender**, ***n*** **(%)**					0.200
Male	169 (68.4)	32 (60.4)	16 (61.5)	121 (72.0)	
Female	78 (31.6)	21 (39.6)	10 (38.5)	47 (28.0)	
**Urban residents**, ***n*** **(%)**					0.695
No	116 (48.3)	24 (48.0)	10 (40.0)	82 (49.7)	
Yes	124 (51.7)	26 (52.0)	15 (60.0)	83 (50.3)	
**Region**, ***n*** **(%)**					0.474
From shanghai	49 (19.8)	13 (24.5)	6 (23.1)	30 (17.9)	
Not from Shanghai	198 (80.2)	40 (75.5)	20 (76.9)	138 (82.1)	
**Annual disposable household income per capita (CNY)**, ***n*** **(%)**					0.905
Below 50K	72 (29.5)	14 (26.9)	8 (30.8)	50 (30.1)	
Above 50K	172 (70.5)	38 (73.1)	18 (69.2)	116 (69.9)	
**Disease severity**, ***n*** **(%)**					0.044^*^
LR	97 (39.3)	27 (50.9)	13 (50.0)	57 (33.9)	
I/HR	150 (60.7)	26 (49.1)	13 (50.0)	111 (66.1)	
**Serious adverse events**^b^ ***n*** **(%)**					0.383
No	243 (98.4)	52 (98.1)	25 (96.2)	166 (98.8)	
Yes	4 (1.6)	1 (1.9)	1 (3.8)	2 (1.2)	
**On steroids**^c^ ***n*** **(%)**					< 0.001^**^
No	87 (35.2)	19 (35.8)	26 (100)	42 (25.0)	
Yes	160 (64.8)	34 (64.2)	0 (0)	126 (75.0)	
**Disease duration (month)**					< 0.001^**^
Mean (SD)	10.9 (±8.75)	1.04 (±0.820)	3.50 (±0.957)	15.2 (±7.41)	
**CHU9D parent-rated utility score**					< 0.001^**^
Mean (SD)	0.79 (±0.17)	0.73 (±0.19)	0.74 (±0.18)	0.82 (±0.16)	

SD, standard deviation; CNY, Chinese Yuan; LR, low risk; I/HR, intermediate /high risk.

* P < 0.05 and

** P < 0.001, respectively.

a Fisher exact test was performed to examine association between two categorical variables; ANOVA was used to test differences in continuous variables among 3 treatment phases.

b Serious adverse events (SAE): having SAE in the preceding 4 weeks.

c On steroids: use of steroids today or in the preceding week.

One hundred fifty participants (60.7%) were at intermediate/high risk (I/HR) according to the treatment protocol. The vast majority of patients (98.4%) had no serious adverse events in the past four weeks, and 64.8% were on steroids in the preceding week. The mean (SD) disease duration since diagnosis was 10.9 (±8.75) months. There are significant differences among three treatment phases in disease severity (*P* = 0.043), on steroids (*P* < 0.001), and disease duration (*P* < 0.001).

Only 11.3% of respondents reported full health (utility score is 1). The score distribution was shown in Supplementary Figure S1. The mean (SD) utility scores were 0.79 (±0.17), 0.73 (±0.19), 0.74 (±0.18), and 0.82 (±0.16) for all the patients, and those at the three treatment phases (induction, consolidation, and maintenance) respectively. There were significant differences in utility scores among the treatment phases (*P* < 0.001).

### Influencing factors of utility scores

Table 2 shows the associations between patient characteristics and utility scores. Age (Pearson's *r* = 0.134*, P* = 0.036) and disease duration (Pearson's *r* = 0.212*, P* < 0.001) were significantly associated with utility scores, while the rest were not.

**Table 2:** Bivariate analysis of associations between patient characteristics and utility scores.

**Patient characteristics**	***N*** **= 247**	**Utility scores**		***P*** **-value^a^**
		**Mean**	**SD**	
**Age**	**247**	**r = 0.134**		0.036^*^
**Disease duration**	247	r = 0.212		< 0.001^**^
**Gender**				0.119
Male	169	0.803	0.162	
Female	78	0.763	0.196	
**Disease severity**				0.201
LR	97	0.773	0.169	
I/HR	150	0.802	0.177	
**Serious adverse events**				0.934
No	243	0.790	0.175	
Yes	4	0.797	0.109	
**On steroids**				0.543
No	87	0.781	0.158	
Yes	160	0.795	0.183	
**Region**				0.813
From shanghai	49	0.785	0.149	
Not from Shanghai	198	0.792	0.180	
**Urban residents**				0.319
No	116	0.803	0.180	
Yes	124	0.780	0.168	
**Annual disposable household income per capita(CNY)**				0.285
Below 50 K	72	0.808	0.169	
Above 50 K	172	0.782	0.176	

SD, standard deviation; CNY, Chinese Yuan; LR, low risk; I/HR, intermediate /high risk.

* P < 0.05 and

** P < 0.001, respectively.

a t-test was performed to examine association between utility scores and each categorical variable; Pearson's r was computed to test association between utility scores and each continuous variable.

Table 3 displays the stepwise multivariable linear regression models of utility scores. Treatment phase remained significant throughout Model 1 to 5. Utility scores during induction (*Beta* = −0.086, *P* = 0.005) and consolidation (*Beta* = −0.074, *P* = 0.043) were constantly lower than those during maintenance even after adjusting for age, gender, disease severity, and income level in Model 5.

**Table 3:** Multiple linear regression analyses of factors associated with CHU9D-CHN utility scores.

**Variables**	**Model 1**			**Model 2**			**Model 3**			**Model 4**			**Model 5**		
	**Beta**	**SE**	***P*** **-value**	**Beta**	**SE**	***P*** **-value**	**Beta**	**SE**	***P*** **-value**	**Beta**	**SE**	***P*** **-value**	**Beta**	**SE**	***P*** **-value**
Intercept	0.819	0.013	< 0.001^**^	0.775	0.031	< 0.001^**^	0.782	0.031	< 0.001^**^	0.781	0.032	< 0.001^**^	0.800	0.038	< 0.001^**^
**Treatment phases**															
Induction	−0.094	0.029	0.001^*^	−0.088	0.030	0.004^*^	−0.084	0.030	0.005^*^	−0.084	0.031	0.007^**^	−0.086	0.031	0.005^*^
Consolidation	−0.081	0.036	0.028^*^	−0.077	0.036	0.033^*^	−0.074	0.036	0.041^*^	−0.073	0.036	0.044^**^	−0.074	0.036	0.043^*^
Maintenance	Ref			Ref			Ref			Ref			Ref		
Age (year)				0.005	0.003	0.097	0.005	0.003	0.086	0.005	0.003	0.124	0.005	0.003	0.153
**Gender**															
Male							Ref			Ref			Ref		
Female							−0.032	0.025	0.205	−0.032	0.025	0.203	−0.029	0.025	0.245
**Disease severity**															
LR										Ref			Ref		
I/HR										0.005	0.024	0.848	0.005	0.025	0.843
**Annual disposable household income per capita (CNY)**															
Below 50K													Ref		
Above 50K													−0.025	0.034	0.300

SE, standard error; CNY, Chinese Yuan; LR, low risk; I/HR, intermediate /high risk.

* P < 0.05 and

** P < 0.001, respectively.

### Responses to CHU9D-CHN by dimensions

**Figure 1:**
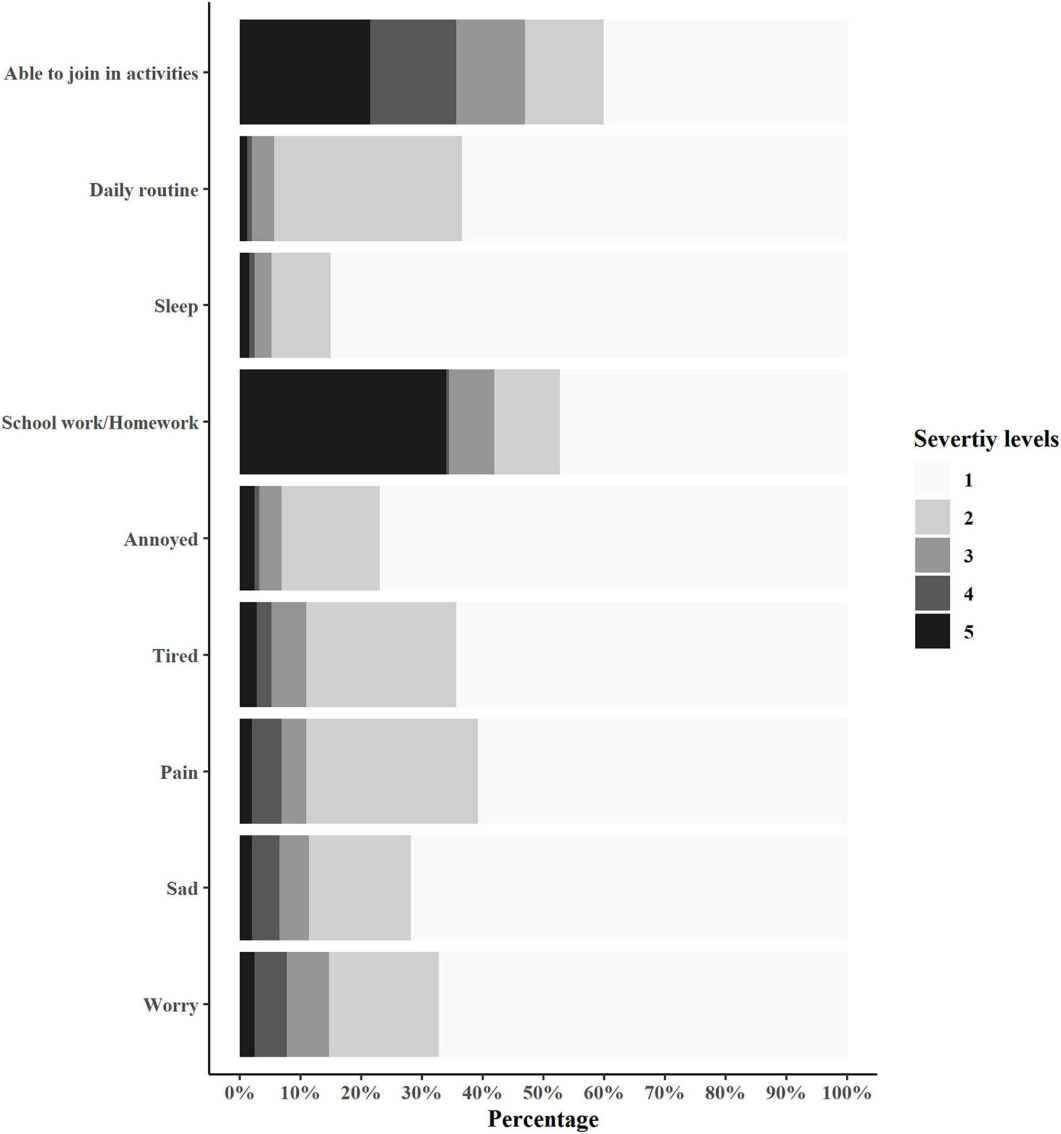
Distribution of responses to each item of CHU9D–CHN.

Figure 1 summarizes the utility scores of patients in 9 dimensions. Less than 3% reported the highest severity level on each of the following 7 dimensions: “worry” (2.46%), “sad” (2.04%), “pain” (2.02%), “tired” (2.83%), “annoyed” (2.43%), “sleep” (1.62%), and “daily routine” (1.22%). More than 30% reported higher severity levels (i.e., Level 4 and 5) on “school work/homework” (34.43%), and “able to join in activities” (35.63%).

Table 4 presents the association between patient characteristics and responses (high vs. low severity level) to “school work/homework” and “able to join in activities” dimensions in bivariate analysis. Lower age and induction phase were found to be significantly associated with high severity reported on the “school work/homework” dimension.

**Table 4:** Bivariate analysis of associations between patient characteristics and responses to “school work/homework” and “able to join in activities” dimensions.

**Variables**	**School work/homework**			**Able to join in activities**		
	**Low**	**High**	***P*** **-value** ^a^	**Low**	**High**	***P*** **-value** ^a^
	**(*****N*** = **158)**	**(*****N*** = **83)**		**(*****N*** = **159)**	**(*****N*** = **88)**	
**Age (year)**			0.002^*^			0.060
Mean (SD)	8.67 (±3.49)	7.24 (±3.30)		7.80 (±3.37)	8.67 (±3.59)	
**School age**			0.001^*^			0.337
Age below 6	45 (28.5)	42 (50.6)		63 (39.6)	29 (33.0)	
Age 6 or above	113 (71.5)	41 (49.4)		96 (60.4)	59 (67.0)	
**Gender**, ***n*** **(%)**			0.772			0.392
Male	107 (67.7)	58 (69.9)		112 (70.4)	57 (64.8)	
Female	51 (32.3)	25 (30.1)		47 (29.6)	31 (35.2)	
**Disease severity**, ***n*** **(%)**			0.330			0.686
LR	57 (36.1)	36 (43.4)		64 (40.3)	33 (37.5)	
I/HR	101 (63.9)	47 (56.6)		95 (59.7)	55 (62.5)	
**Annual disposable household income per capita (CNY)**, ***n*** **(%)**			0.372			0.772
Below 50K	49 (31.4)	21 (25.3)		45 (28.8)	27 (30.7)	
Above 50K	107 (68.6)	62 (74.7)		111 (71.2)	61 (69.3)	
**Treatment phases**, ***n*** **(%)**			< 0.001^**^			0.041^*^
Induction	23 (14.6)	28 (33.7)		28 (17.6)	25 (28.4)	
Consolidation	14 (8.9)	12 (14.5)		14 (8.8)	12 (13.6)	
Maintenance	121 (76.6)	43 (51.8)		117 (73.6)	51 (58.0)	

SD, standard deviation; CNY, Chinese Yuan; LR, low risk; I/HR, intermediate /high risk.

* P < 0.05 and

** P < 0.001, respectively.

a Fisher exact test was performed to examine association between two categorical variables; t-test was used to examine differences in continuous variables between the two severity groups.

Table 5 indicates that induction phase (vs. maintenance, OR = 3.46, *P* < 0.001) and lower school age (OR = 2.38, *P* = 0.004) were independently associated with the high severity level reported on the “school work/homework” dimension, after adjusting for age, gender, disease severity, and income level. Only the induction phase (vs. maintenance, OR = 2.24, *P* = 0.016) was independently associated with the high severity level reported on the “able to join in activities” dimension.

**Table 5:** Multivariable logistic regression analyses of factors associated with high severity levels reported in two CHU9D-CHN dimensions.

**Variables**	**School work/homework**		**Able to join in activities**	
	**Odds ratio (95%CI)**	***P*** **-value**	**Odds ratio (95%CI)**	***P*** **-value**
**Treatment phases**				
Induction	3.46 (1.75–6.92)	< 0.001^**^	2.24 (1.16–4.34)	0.016^*^
Consolidation	2.19 (0.90–5.27)	0.081	2.10 (0.88–4.97)	0.090
Maintenance	Ref		Ref	
**School year**				
Age below 6	2.38 (1.32–4.17)	0.004^*^	0.66 (0.37–1.18)	0.162
Age 6 or above	Ref		Ref	
**Gender**				
Male	Ref		Ref	
Female	0.77 (0.41–1.42)	0.414	1.18 (0.66–2.08)	0.579
**Disease severity**				
LR	Ref		Ref	
I/HR	1.01 (0.56–1.83)	0.981	1.16 (0.66–2.06)	0.612
**Annual disposable household income per capita (CNY)**				
Below 50K	Ref		Ref	
Above 50K	1.34 (0.72–2.58)	0.363	0.91 (0.51–1.65)	0.755

CNY, Chinese Yuan; LR, low risk; I/HR, intermediate /high risk.

* P < 0.05 and

** P < 0.001, respectively.

## Discussion

This is the first study that measured the health utility of children with acute lymphoblastic leukemia in China, and evaluated utility weights of different treatment phases. The mean health utility score was 0.79 (SD = 0.17) for all children with pALL in our sample, approaching the lower end of the range (0.78–0.92) from previous studies that reported self-rated CHU9D scores of health Chinese children (23, 24, 29–32). The mean health utility scores were 0.73 (SD = 0.19) for those in induction, and 0.74 (SD = 0.18) for consolidation. Both were significantly lower than that in maintenance (mean = 0.82, SD = 0.16).

### Impact of treatment phase on CHU9D utility scores

Treatment phase was found to be an independent influencing factor of health utility after adjusting for socioeconomic factors. Patients during induction seemed to have the lowest health utility. This finding was not surprising, because patients tend to experience the most pain and discomfort from adverse effects and/or complications during the induction phase whereby the chemotherapy was most intensive. Meanwhile, their primary caregivers (usually parents) were most overwhelmed by what patients suffered from the chemotherapy during this very initial stage of treatment. When it came to the second phase—consolidation, both patients and their caregivers were likely to be less overwhelmed and slowly getting used to the cancer treatment routines, contributing to slightly higher utility values. In the last phase—maintenance, as the treatment intensity went lower and patient condition became more stable, their health state would be largely improved, leading to the highest health utility values.

This trend was generally consistent with the previous studies (15–17), where health utility values increased as treatment began from the induction phase, through CNS and intensification, to the continuation phase as defined by different treatment protocols. To enable a fair comparison, their CNS, intensification and continuation phases were considered equivalent to our consolidation, early (week 16–35) and late (week 36–125) maintenance phases respectively, after a careful examination of relevant treatment protocols by experienced clinicians. More specifically, our mean utility score of the induction phase (0.73) was well within the range reported by the 3 previous studies (0.66–0.81). The same finding was also shown when comparing those for the maintenance phase (i.e., 0.82 vs. 0.78–0.91).

However, for the consolidation phase, our estimate (0.74) was slightly lower than those presented by Furlong and colleagues (0.75–0.90) (15–17). A plausible explanation could be that we chose to use CHU9D, a very different utility instrument from HUI2/3 adopted by all the previous 3 studies. HUI2 and HUI3 comprise 7 and 8 dimensions respectively, covering sensation, self-care, fertility, vision, hearing, speech, ambulation/mobility, dexterity, emotion, cognition, and pain in total. Clearly, both HUI2/3 and CHU9D measure physical, mental and cognitive functions, but only CHU9D contains social function assessed by the dimension “able to join in activities.” It was quite common for pALL patients who were undergoing active treatment to feel unable to join in routine activities with their peers due to frequent hospitalizations, hospital visits and/or weak physical condition. Therefore, without measuring such an important aspect of impact on HRQoL, HUI2/3 would probably over-estimate utility values of some health states at baseline and consequently under-estimate the true effect of an intervention that aims to enhance HRQoL. These results suggested that the use of HUI2/3, as opposed to CHU9D, in cost-utility analysis may lead to less favorable estimates of incremental effects and consequently less attractive incremental cost-effectiveness ratios (ICERs) with lower chance of adopting more expensive but also more effective treatment alternatives. Nonetheless, due to the absence of head-to-head comparison between HUI2/3 and CHU9D, it clearly calls for primary studies to provide empirical evidence to elucidate this issue. More importantly, it is suggested that the choice of a preference-based HRQoL instruments for economic evaluations should be carefully justified, as it may have an important impact on the decision making based on the results of such evaluations.

### Lack of association between disease severity and CHU9D utility scores

Unexpectedly, disease severity was found not associated with health utility regardless of adjustment for other influencing factors. Unlike other types of childhood cancer (10, 33), disease severity of pALL was not so much about symptoms or clinical presentation, but rather determined by molecular measurements in order to guide treatment. Nevertheless, patients with intermediate or high-risk ALL received more intensive chemotherapy than those with low risk mainly during induction and consolidation phases, hence they were more likely to have adverse events and consequently lower health utility. However, our data showed otherwise. This is not entirely consistent with previous studies either. In fact, only one of the previous pALL studies investigated the association between disease severity and health utilities. Rae and colleagues (16) found that it was only during intensification that the high-risk group had significantly lower utility scores for both HUI2 and HUI3, but there were no significant differences between groups during induction, CNS therapy, or continuation, nor at 2 years after treatment. They believed that the plausible reason could be during this stage of therapy, only high-risk patients were given doxorubicin and 3-fold higher doses of steroids that are commonly associated with nausea/vomiting and mucositis. In contrast, a possible explanation of our finding could be that treatment at any phases was well tolerated in our study sample as only very few patients (*n* = 4, 1.6%) reported serious adverse effects, thus the perceived health status of patients might be quite similar, regardless of their disease severity.

### Most impaired CHU9D dimensions

Moreover, among the 9 dimensions of CHU9D-CHN, our patients were found to have more difficulties in “school work/homework,” and “able to join in activities” dimensions, especially in the induction phase. Due to more frequent hospitalizations and intensive treatment, patients in the induction phase were expected to have less social contact, combined with physical and psychological discomfort at the beginning of treatment, consequently accomplish less in routine learning activities and participate less in social activities (34). Making it even worse, ever since the COVID-19 pandemic outbreak, due to ward visiting restrictions, ward volunteers were no longer allowed to access and thus had to cease our routine “sunshine care” program, which used to provide a variety of engaging activities specially designed for sick children such as playing board games, reading or general conversation for companionship and support. Without access to “sunshine care,” our patients had fewer opportunities to take part in any activities during hospitalization, making the difference between induction and maintenance phases more obvious. Therefore, we call for healthcare providers, social workers, charitable organizations and other stakeholders to collaborate for developing a virtual version of “sunshine care” to provide live, online patient support, unbound by restrictions of time or location. In the future, it is hoped that “sunshine care” and similar in-person support programs be implemented for all hospitalized children with cancer. More research is also encouraged to explore the effect of other innovative interventions such as tailored physical activities (35–37) and art therapy (38) in enhancing HRQoL of pALL patients.

Another interesting finding is that our data showed preschool children were more likely to experience difficulties in the dimension of “school work/homework,” than school-age children. While all the previous utility-based studies (14–17) found no association between age and HRQoL, more equivocal evidence emerged regarding child age from non-utility studies (11). In general, most of them reported either no association or an association between increasing age of a child and worse overall HRQL and/or specific HRQL domains including school and cognition subscales (11). Our seemingly counter-intuitive result in the “school work/homework” dimension might be due to the lack of clarity in the description of this particular CHU9D-CHN item. The item is phrased as “school work/homework (e.g., reading a book, writing, undertaking assignments)” by literal translation. In China, preschool children do not usually have formal “school work/homework” especially in the form of reading or writing assignments. So it is very likely that some Chinese parents might perceive this item to be irrelevant on the first sight, and thus had mistaken the last response level “can't do” for “not applicable” without a second thought. While this speculation is now being investigated in our follow-up study, researchers are strongly encouraged to pay extra attention to this particular item in a pilot testing of CHU9D-CHN to avoid potential misconceptions. Another suggestion would be adding more examples to this item, such as “reading picture books, drawing or singing songs/chants,” to help parents of young preschool children relate to their routine learning activities.

### Study limitations

Last but not least, our results need to be interpreted in light of several study limitations. First, a major limitation is that our patients were recruited exclusively from a designated national children's medical center—a well-known center of excellence for pediatric diseases, which may limit the study's generalizability to all pALL patients in China as it is more vulnerable to selection bias than population-based studies. The center is located in Shanghai, yet serves most provinces and cities in China and patients outside the city account for more than 70%. The selection bias favoring more severely impaired patients and/or patients from more affluent families cannot be excluded. Indeed, our patients may be from a higher socioeconomic class as suggested by their income level and willingness to seek the best specialist healthcare. Nevertheless, our sample consists of about 80% of patients outside Shanghai, 50% from rural areas, still representing a good catchment. Second, we cannot exclude residual confounding effects by unmeasured variables (e.g., family functioning level, undergoing lumbar punctures on the day of survey) in the multivariate analyses. However, we did examine treatment phases which provide comprehensive information about the treatment intensity. Third, the cross-sectional design of our study does not provide any information on changes in HRQoL as the disease progresses. Forth, despite that CHU9D is so far the only utility instrument for children with a China-specific value set, it should be noted that the value set was derived from Chinese students aged 8–17 and thus may not represent preference of the general population, or specifically, the taxpayers in China. Future studies are encouraged to investigate this speculation by developing general population-based value sets for CHU9D or other child-friendly instruments and then conducting head-to-head comparative analyses. Lastly, compared with self-rated utility scores, caregivers may overestimate or underestimate how children perceived their own HRQoL as suggested by some studies (39–41). Nonetheless, caregiver-rated utility scores were probably the best available data for our sample as about 56% were < 8 years old and didn't have adequate reading and comprehension abilities to complete the survey. More work is warranted in the future to collect and then compare utility scores from both eligible pALL children and their parents for clarifying any bias that caregiver ratings may introduce.

## Conclusion

In conclusion, our study measured for the first time health utility of Chinese children with pALL. The utility scores may enable comparisons using QALYs across different clinical populations to inform decision makers for establishing healthcare priorities. We examined health utility weights of different treatment phases, providing important inputs into economic models that are utilized commonly for health resource allocations. Our results also add valuable information to the scarce evidence on factors that influence caregiver ratings of patient preferences in the area of pALL. Moreover, “school work/homework” and “able to join in activities” were identified as the most impaired aspects of HRQoL. These can have meaningful implications in developing appropriately tailored interventions to improve patient support and ultimately HRQoL for children with pALL in China.

## Data availability statement

The original contributions presented in the study are included in the article/Supplementary material, further inquiries can be directed to the corresponding authors.

## Ethics statement

The studies involving human participants were reviewed and approved by the Ethics Committee of Shanghai Children's Medical Center. Written informed consent to participate in this study was provided by the participants' legal guardian/next of kin.

## Author contributions

WW and JC contributed to conception and design of the study. MJ and XZ led the data collection. YD and WW performed the statistical analysis and wrote the first draft of the manuscript. JC and XZ contributed to interpretation of data. All authors contributed to critical revision of the manuscript for important intellectual content, read, and approved the final version.
