# Supplementary figures and images for: Health utility of children with acute lymphoblastic leukemia in China
Source: Front Public Health. 2023 Jan 4;10:1069336. doi: 10.3389/fpubh.2022.1069336 (PMC9846596; doi:10.3389/fpubh.2022.1069336)

Supplemental Figure 1. CHU9D utility score distribution

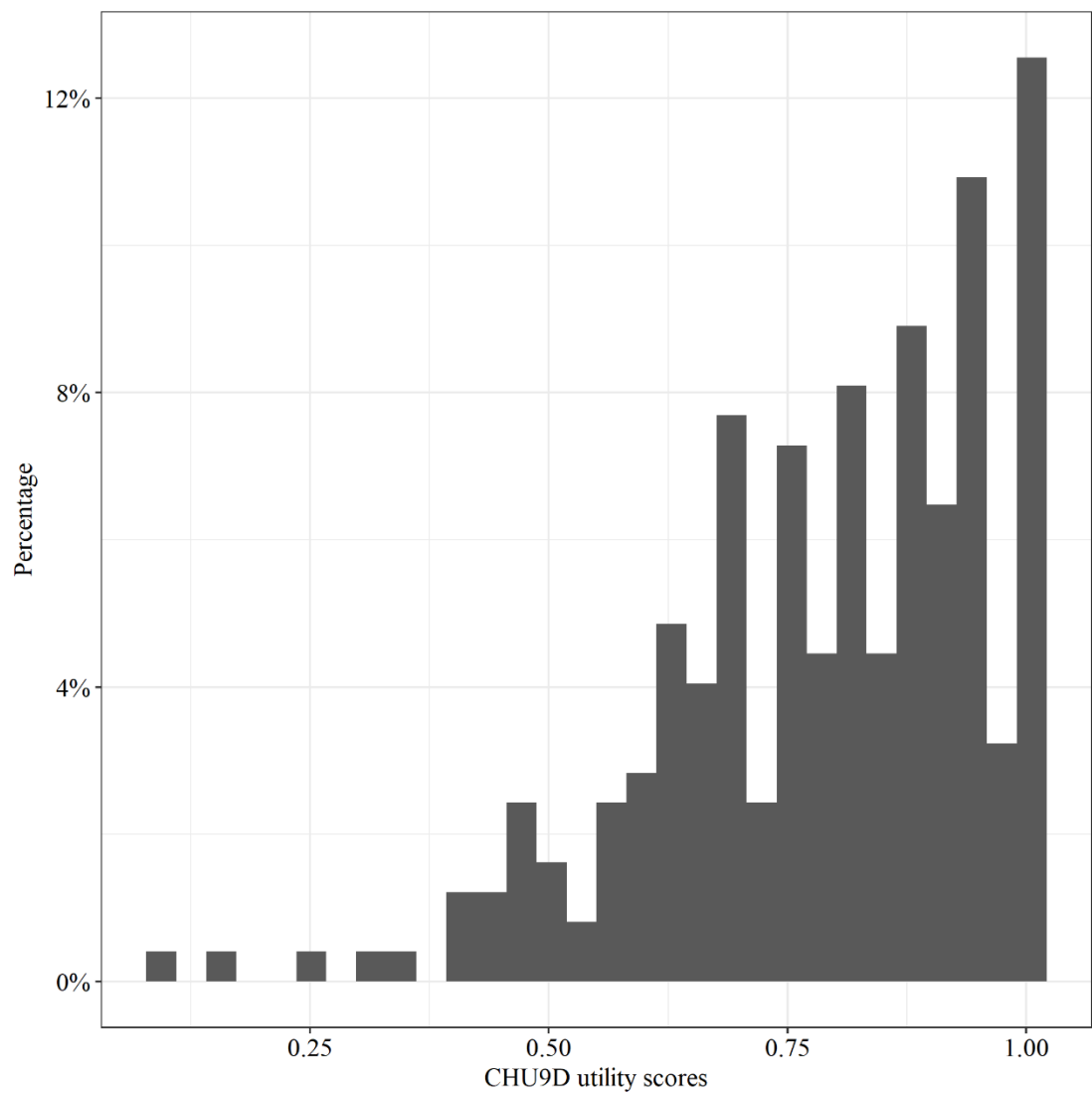

Supplement: Supplementary file 1 [file Image_1.pdf]
